# Transcriptomics-based screen for genes induced by flagellin and repressed by pathogen effectors identifies a cell wall-associated kinase involved in plant immunity

**DOI:** 10.1186/gb-2013-14-12-r139

**Published:** 2013-12-20

**Authors:** Hernan G Rosli, Yi Zheng, Marina A Pombo, Silin Zhong, Aureliano Bombarely, Zhangjun Fei, Alan Collmer, Gregory B Martin

**Affiliations:** 1 Boyce Thompson Institute for Plant Research, Ithaca, NY 14853, USA; 2 Instituto de Investigaciones Biotecnológicas – Instituto Tecnológico de Chascomús (IIB-INTECH) UNSAM-CONICET, Chascomús B7130IWA, Buenos Aires, Argentina; 3 School of Life Sciences, The Chinese University of Hong Kong, NT, Hong Kong; 4 Department of Plant Pathology and Plant-Microbe Biology, Cornell University, Ithaca, NY 14853, USA; 5 Department of Biological Sciences, King Abdulaziz University, Jeddah 21589, Saudi Arabia

## Abstract

**Background:**

Microbe-associated molecular patterns, such as those present in bacterial flagellin, are powerful inducers of the innate immune response in plants. Successful pathogens deliver virulence proteins, termed effectors, into the plant cell where they can interfere with the immune response and promote disease. Engineering the plant immune system to enhance disease resistance requires a thorough understanding of its components.

**Results:**

We describe a high-throughput screen, using RNA sequencing and virus-induced gene silencing, to identify tomato genes whose expression is enhanced by the flagellin microbe-associated molecular pattern flgII-28, but reduced by activities of the *Pseudomonas syringae* pv. *tomato* (*Pst*) type III effectors AvrPto and AvrPtoB. Gene ontology terms for this category of *Flagellin-induced repressed by effectors* (*FIRE*) genes showed enrichment for genes encoding certain subfamilies of protein kinases and transcription factors. At least 25 of the *FIRE* genes have been implicated previously in plant immunity. Of the 92 protein kinase-encoding *FIRE* genes, 33 were subjected to virus-induced gene silencing and their involvement in pattern-triggered immunity was tested with a leaf-based assay. Silencing of one *FIRE* gene, which encodes the cell wall-associated kinase SlWAK1, compromised the plant immune response resulting in increased growth of *Pst* and enhanced disease symptoms.

**Conclusions:**

Our transcriptomic approach identifies *FIRE* genes that represent a pathogen-defined core set of immune-related genes. The analysis of this set of candidate genes led to the discovery of a cell wall-associated kinase that participates in plant defense. The *FIRE* genes will be useful for further elucidation of the plant immune system.

## Background

The plant immune system involves two related inducible responses. The first response is activated by the detection of microbe-associated molecular patterns (MAMPs) by the extracellular domains of pattern recognition receptors (PRRs) [1]. A small number of PRRs have been identified in plants that recognize MAMPs derived from flagellin, elongation factor Tu, an ethylene-inducing xylanase, and certain non-proteinaceous MAMPs [2]. Additionally, damage-associated molecular patterns (DAMPs), which typically appear in the apoplast as a consequence of pathogen attack, function as host-derived elicitors [3]. The activation of pattern-triggered immunity (PTI) by MAMPs and DAMPs leads to changes in the intracellular calcium concentration, production of reactive oxygen species, activation of mitogen-activated protein kinase (MAPK) cascades and transcriptional reprogramming [4]. These events lead, in a largely unknown manner, to inhibition of pathogen growth and suppression of disease. Successful pathogens deliver virulence proteins (effectors) into the plant cell and a majority of these proteins appear to function by interfering with host immunity-associated events triggered by MAMP recognition [5]. A second plant defense response, effector-triggered immunity (ETI), can be activated in cases where a specific pathogen effector or its activity is recognized by a host nucleotide-binding leucine-rich repeat-containing (NB-LRR) resistance protein [1].

The interaction of tomato with *Pseudomonas syringae* pv. *tomato* (*Pst*) is a powerful model system for understanding the molecular basis of bacterial pathogenesis and plant immunity [6]. *Pst* enters the apoplastic space of tomato leaves through stomata or wound sites. The plant responds to *Pst* even at the stomatal entry stage, although the bacterium can overcome this response with the aid of the phytotoxin coronatine [7]. *Pst* present in the apoplast is detected by PRR-mediated recognition of various MAMPs thereby activating PTI. The best characterized of these MAMPs is a 22-amino acid region of flagellin (flg22), which is recognized by FLS2, a PRR with extracellular leucine-rich repeats (LRRs) and an intracellular protein kinase domain [8]. The mechanism by which FLS2 activates intracellular events involves another LRR receptor-like kinase, BAK1, as well as cytoplasmic protein kinases of the PBS1-like family [9-11]. Recently, another MAMP derived from flagellin, flgII-28, has been identified [12]. The PRR that detects flgII-28 is unknown, but it appears to play an important role in activating PTI in solanaceous species [13].

Two *Pst* effector proteins, AvrPto and AvrPtoB, have been studied extensively and found to play multiple important roles during the tomato–*Pst* interaction [14]. These roles are partially redundant and deletion of both effector genes is required before a marked decrease in virulence is observed. Some of this redundancy is attributable to the fact that each effector binds and interferes with protein kinase domains of the FLS2–BAK1 complex, thereby disrupting the host response to flg22 [14]. However, each effector targets additional host proteins using independent domains found in each effector [15,16]. In a well-studied example of ETI, the host protein kinases Fen and Pto appear to act as decoys of the real kinase virulence targets. Interaction of AvrPto and AvrPtoB with these decoys triggers a host immune response mediated through the NB-LRR protein Prf [17].

Host responses associated with PTI and ETI are complex and have been studied by both reductionist approaches focused on individual components and mechanisms as well as by large-scale systems biology approaches looking at the totality of metabolomic, proteomic and transcriptomic changes. Previous transcriptomic studies in *Arabidopsis thaliana*, tomato and other species have relied primarily on microarrays and cDNA-amplified fragment length polymorphism (AFLP) methods, which, though limited in their dynamic range and, in some cases, in their genome coverage, have yielded important insights into PTI, ETI and the role of pathogen effectors in suppressing the former responses [18-25]. However, the emergence of RNA sequencing (RNA-seq) and associated statistical analyses as well as the availability of improved gene annotation now allows for the development of a much more comprehensive view of subtle gene expression changes, and how pathogen effectors impact these changes as part of their immunity-suppressive activities. Here we have taken advantage of the recently completed tomato genome sequence [26], the comprehensive nature of RNA-seq analysis, virus-induced gene silencing (VIGS) and the well-characterized tomato–*Pst* system to develop a genome-wide screen for genes whose expression is specifically altered in response to both MAMP detection and subsequent activities of pathogen effectors.

## Results

### Development of an RNA sequencing approach to examine the plant response to MAMPs present in various bacterial pathogens and non-pathogens

To characterize the PTI response of tomato and the effect of the delivery of a subset of effectors, we performed an RNA-seq analysis of tomato Rio Grande *prf3* leaves challenged with either the flgII-28 peptide or the bacterial strains described in Table 1. We focused on flgII-28 because it has recently emerged as a second MAMP derived from flagellin, in addition to flg22, which is perceived specifically by solanaceous species [12,13]. In this case we chose 1 μM concentration, which is the same as the lowest amount used for flg22 treatments in previous microarray studies of *Arabidopsis*[19,23]. Using *Pseudomonas fluorescens*, *Pseudomonas putida* and flgII-28 we investigated PTI-associated transcriptional changes, while *Agrobacterium tumefaciens* and *Pseudomonas syringae* pv. *tomato* (*Pst*) strain DC3000Δ*hrcQ-U*Δ*fliC* were chosen to examine the effects due to MAMPs other than flagellin. The comparison between the response to DC3000 and the mutant DC3000Δ*avrPto*Δ*avrPtoB* was designed to uncover the effect of two ‘early-acting’ type III effectors [27]. We chose a sampling time (6 h) and a bacterial titer (5 × 10^6^ cfu/mL) to capture early PTI-associated transcriptional changes and also the effects of early-acting effectors on these changes. For both the non-pathogenic *Pseudomonas* species and *A. tumefaciens* we used the same time but a higher titer (10^8^ cfu/mL) to maximize the plant response.

**Table 1:** Details of the treatments used for RNA sequencing analysis of the tomato immune response

**Treatment**	**Concentration**	**Comment**
flgII-28	1 μM	28-amino acid peptide from *Pseudomonas syringae* pv. *tomato* T1
*Pseudomonas syringae* pv. *tomato* (*Pst*) DC3000	5 × 10^6^ cfu/mL	Wild-type *Pst* strain
DC3000Δ*hrcQ-U*Δ*fliC*	5 × 10^6^ cfu/mL	DC3000 mutant, lacking a functional T3SS and flagellin
DC3000Δ*avrPto*Δ*avrPtoB*	5 × 10^6^ cfu/mL	DC3000 mutant, with a functional T3SS, but lacking *avrPto* and *avrPtoB*
*Pseudomonas fluorescens* 55	10^8^ cfu/mL	Soil bacterium
*Pseudomonas putida* KT2240	10^8^ cfu/mL	Soil bacterium
*Agrobacterium tumefaciens* GV2260	10^8^ cfu/mL	Disarmed plant pathogen
MgCl_2_	10 mM	Mock treatment
**-**	**-**	Untreated plants

T3SS: type III secretion system; cfu: colony-forming units.

The RNA-seq analysis over all treatments revealed reads per kilobase of exon model per million mapped reads (RPKM) ranging from 0 to approximately 39,000 for the 34,727 genes predicted in tomato [26]. We focused on genes with ≥3 RPKM in at least one treatment based on analysis of the RPKM of genes known to play a role in immunity (Additional file 1: Figure S1). We refer to this group of 17,597 genes as the expressed genes. All of our RNA-seq data are available from the Tomato Functional Genomics Database [28] under accession no. D007.

### Flagellin-derived peptides are the major MAMPs causing immunity-associated transcriptional changes in tomato

**Figure 1:**
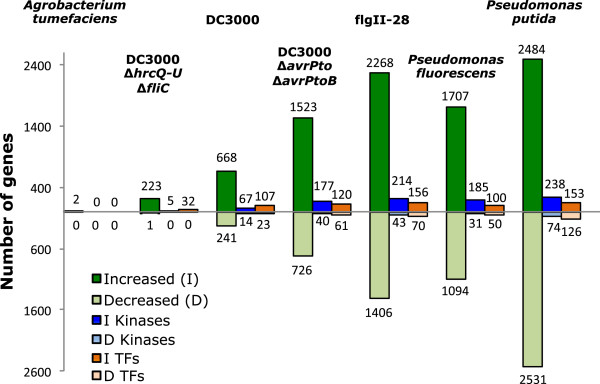
**Flagellin contains the major MAMPs impacting transcriptional reprogramming following *Pseudomonas syringae *infiltration into tomato leaves.** Shown is the total number of genes, protein kinases (kinases) and transcription factors (TFs) with increased (I) or decreased (D) transcript abundance compared to the mock, 6 h after treatment. A ≥2-fold difference and *P* < 0.05 were used as cut-offs. The number of genes in each category is also shown. See Table 1 for details of the treatments.

A first analysis of the genes whose transcript abundance was affected by the treatments (Figure 1) revealed two main groups based on the overall change compared to the mock treatment (≥2 fold and *P* < 0.05). One group had a small number of genes (<250; *A. tumefaciens*, DC3000Δ*hrcQ-U*Δ*fliC*) while the other had a much larger number (>900; all the remaining treatments). These two extreme responses were associated with the presence or absence of a ‘perceivable’ flagellin (FliC protein) in the treatment. FliC in *A. tumefaciens* is divergent from other flagellin proteins perceived by plants, with a non-active flg22 region and probably a non-perceivable flgII-28 region due to a lack of conserved amino acids in this region (Additional file 2: Figure S2). The DC3000Δ*hrcQ-U*Δ*fliC* mutant lacks flagellin but, as with *A. tumefaciens*, would still have other MAMPs. However, our results indicate that in tomato other MAMPs play a minor role in gene expression changes observed at 6 h. A common feature in the second group of treatments is the presence of flagellin or a portion thereof (flgII-28). In general, for these treatments more genes are induced than suppressed, and this trend was also observed when protein kinase and transcription factor (TF) genes were analyzed separately (Figure 1). Even though the titers used for *P. fluorescens* and *P. putida* were the same, the number of genes affected by *P. putida* was higher, suggesting a more robust PTI response similar to that observed previously in *Nicotiana benthamiana*[29]. There are 1,148 protein kinase genes predicted in tomato (3.3% of the 34,727 gene models [30]). In our treatments, protein kinase genes represented between 7% and 11% of the induced genes, indicating an enrichment similar to that observed in *Arabidopsis* upon flg22 treatment [19].

**Figure 2:**
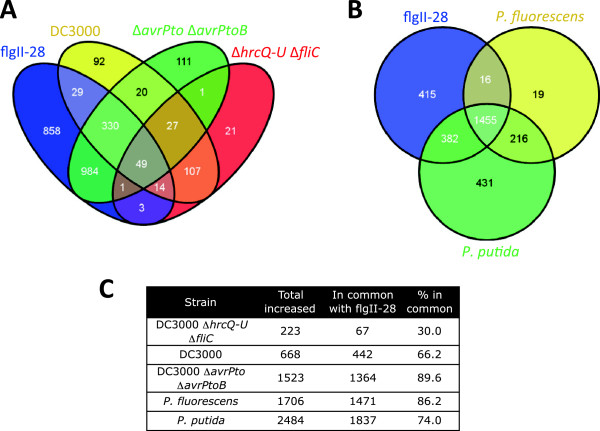
**Overlap between genes induced by bacterial strains and flgII-28 treatment. (A)** Overlap between *Pseudomonas syringae* pv. *tomato* strains and flgII-28; **(B)** Overlap between soil *Pseudomonas* and flgII-28. **(C)** Degree of overlap shown as percentage of genes.

We found there was high degree of overlap in the genes whose expression changed in response to the flgII-28 peptide and different bacterial strains with perceivable flagellin: DC3000Δ*avrPto*Δ*avrPtoB* (90% overlap), *P. fluorescens* (86%) and *P. putida* (74%) (Figure 2, see Additional file 3: Figure S3 for overlap of suppressed genes). This observation, and the fact that we found very little transcriptional reprogramming due to DC3000Δ*hrcQ-U*Δ*fliC* infiltration indicates that flagellin-derived MAMPs (flg22 and flgII-28) have the greatest impact on tomato gene expression changes at the 6 h time point.

### The activities of AvrPto and AvrPtoB counteract a subset of the transcriptional changes that occur upon flagellin perception

**Figure 3:**
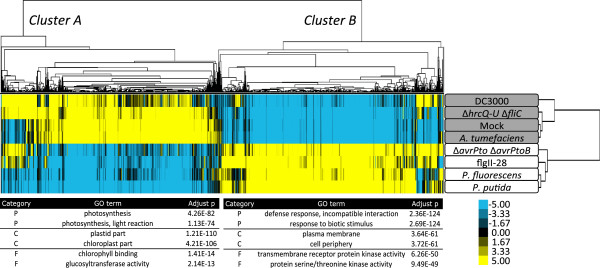
**Clustering analysis reveals that AvrPto and AvrPtoB repress part of the transcriptional reprogramming that is a consequence of flagellin perception.** Hierarchical clustering was based on genes and treatments. From the 17,597 genes expressed in leaves, those with a ratio max/min ≥3 over the various treatments were included in the analysis. Clusters A and B are enriched in genes decreased and increased by flagellin perception, respectively. The color scale shows the deviation from the median for each gene. The top two most enriched GO terms in the categories process (P), component (C) and function (F) are listed below each gene cluster. *A.*, *Agrobacterium*; GO, gene ontology; *P.*, *Pseudomonas*.

We next performed a clustering analysis of both treatments and genes. For this purpose we focused on the 4,150 genes that had ≥3-fold difference between the maximum and the minimum RPKM observed for any two treatments. The genes grouped into two large clusters (Figure 3) each with a similar number of genes, with cluster A encompassing genes having decreased transcript abundance for flagellin-associated treatments and cluster B having genes with the opposite response. Cluster A is enriched in gene ontology (GO) terms such as photosynthesis, chloroplast and chlorophyll binding (Figure 3, see Additional file 4: Table S1 for a complete list of enriched terms). In contrast, cluster B is enriched in terms associated with defense response, plasma membrane and transmembrane receptor protein kinase activity.

The treatments also grouped into two distinct clusters (Figure 3). One cluster included treatments associated with flagellin perception (flgII-28, DC3000Δ*avrPto*Δ*avrPtoB*, *P. fluorescens* and *P. putida*) and the other consisted of ‘flagellin-independent’ treatments (mock, *A. tumefaciens* and DC3000Δ*hrcQ-U*Δ*fliC*). Interestingly, DC3000, which expresses a perceivable flagellin, clustered with this second group. This observation is consistent with the known role of AvrPto and AvrPtoB in interfering with FLS2 and BAK1, components of flagellin perception [14], and raised the possibility that the suppressed genes play particularly important roles in the plant defense response.

### The subset of genes induced by flgII-28 but suppressed by AvrPto/AvrPtoB is enriched in gene ontology terms associated with the cell periphery, biotic interactions and signal transduction

We focused on the set of genes whose expression is increased by flgII-28 and is higher in DC3000Δ*avrPto*Δ*avrPtoB* compared to DC3000 treatment. These are genes for which AvrPto and AvrPtoB activity appears to counteract the transcriptional increase due to flagellin perception. We refer to these as *FIRE* genes (for flagellin-induced, repressed by effectors). From 2,268 genes that are increased by flgII-28 treatment (Figure 1), 622 are *FIRE* genes (≥2 fold and *P* < 0.05; see Additional file 5: Table S2).

A GO term analysis of these 622 *FIRE* genes using the tomato genome as a reference would not be informative, since they derive from a list of genes induced by flgII-28 and are therefore already enriched in immunity-related terms as described for cluster B (Figure 3). For this reason we used the 2,268 genes induced by flgII-28 as the reference. With this approach we found that the *FIRE* genes are enriched, even within the flgII-28-induced gene subset, for immune-related terms and particularly for protein kinases and transmembrane receptors (Additional file 6: Table S3). A total of 92 protein kinase-encoding genes are among the *FIRE* genes including many that have a known role in the immune response: *FLS2.2*, *Bti9*, *RIN4.1*, *RIN4.2*, *Pti4* and *Lectin RLK VI.2* (Additional file 5: Table S2). Furthermore, if we had used a less stringent cut-off for defining the *FIRE* gene list (≥1.5 fold change and *P* < 0.05) we would have included eight additional immunity-related genes such as *SOBIR1*, *SAG101* and *TFT7* (See Additional file 5: Table S2 for a complete list of genes).

**Figure 4:**
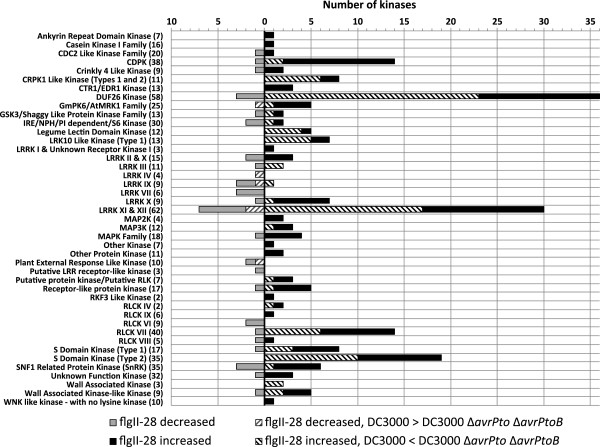
**FlgII-28 perception and the activity of AvrPto and AvrPtoB affects gene expression of certain protein kinase families.** Protein kinase families are listed on the left with the number of expressed members between parentheses. A ≥2-fold difference and *P* < 0.05 were used as cut-offs.

Given the known importance of protein kinases in the immune response, we investigated the families that were affected by our treatments (Figure 4). From the 67 protein kinase families present in tomato [30], 43 were affected by flgII-28 treatment. Some families contained only genes induced by flgII-28 (for example, ankyrin repeat kinases, CTR1/EDR1, MAP2K and MAP3K) and others contained only genes with decreased expression in response to flgII-28 (for example, LRRK IV and VII and RLCK VI). Over 40% of the kinase genes increased by flgII-28 (92/214) were found to be *FIRE* genes.

### Silencing of a *FIRE* gene encoding a cell wall-associated kinase (*SlWAK1*) compromises pattern-triggered immunity

**Figure 5:**
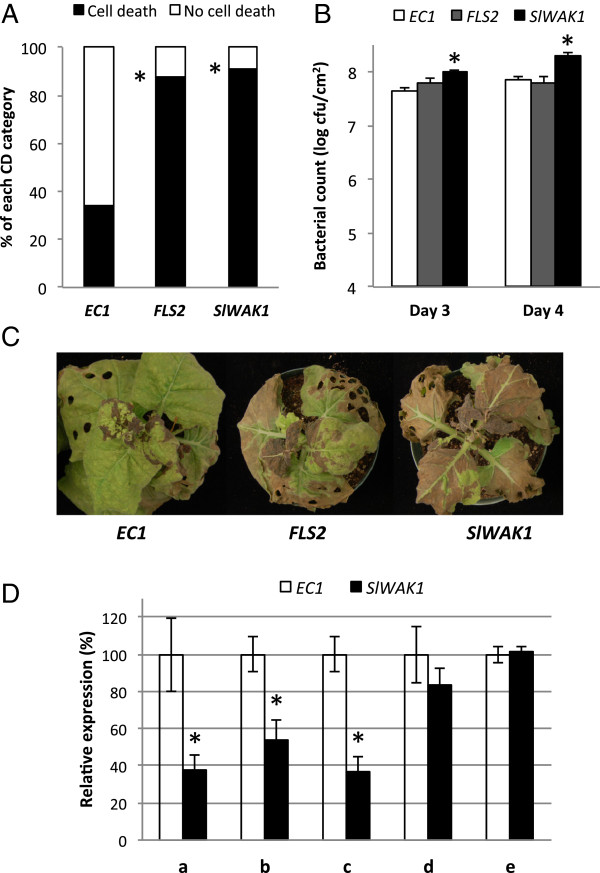
**VIGS silencing of *SlWAK1 *in *Nicotiana benthamiana *results in compromised PTI and increased susceptibility to *Pst*. (A)** Cell death suppression assay. Silenced plants were induced by syringe infiltration of *Pseudomonas fluorescens* and challenged 8 h later with DC3000 in an overlapping area. Cell death symptoms were scored in the overlapping area, on four independently silenced plants with four infiltration sites on two different leaves. Results shown are from 7 days after infiltration (dai). Asterisks indicate significant differences based on Dunnett’s method, using *EC1* as a control (*P* < 0.01). **(B)***Pst* growth in leaves. Silenced plants were vacuum-infiltrated with *P. fluorescens*, inoculated 6 h later with DC3000Δ*hopQ1-1* and sampled to measure bacterial populations. Results shown are the average of six plants per construct with the standard error. Asterisks indicate significant differences based on Dunnett’s method, using *EC1* as a control (*P* < 0.01). **(C)** Disease symptoms of plants treated as described in **(B)** 7 dai. **(D)** Silencing efficiencies in *EC1* and *SlWAK1* silenced plants assessed by qRT-PCR. Silencing efficiency is shown as relative expression compared to the *EC1* control. Data were generated using *EF1α* as reference gene and similar results were obtained using *PP2A*. Data from putative targets: (a) NbS00011055g0005.1, (b) NbS00011055g0014.1 and (c) NbS00011055g0002.1, and a predicted non-target (d) NbS00016938g0011.1 are shown. *PP2A* (e) was used as a cDNA integrity control. Asterisks indicate significant differences using a Student’s *t*-test (*P* < 0.01). dai, days after infiltration; qRT-PCR, quantitative reverse transcription-PCR; *Pst*, *Pseudomonas syringae* pv. *tomato*; PTI, pattern-triggered immunity; VIGS, virus-induced gene silencing.

To investigate the possible contribution to immunity of *FIRE* genes encoding protein kinases, we selected 33 such genes, including at least one member of each kinase category, for analysis using virus-induced gene silencing (VIGS) in *N. benthamiana* (Additional file 5: Table S2). Silenced plants were subjected to a cell death suppression assay (CDSA) [29] to test the involvement of the candidate gene in PTI. Briefly this assay consists of an initial infiltration of *P. fluorescens* as an inducer of PTI, then 8 h later the infiltration of DC3000 (partially overlapping with the first infiltration) as a challenger of this induction. If silencing of a candidate gene results in a faster breakdown of *P. fluorescens* protection, this is an indication that the gene contributes to PTI. VIGS constructs containing a gene fragment from *FLS2* or an *Escherichia coli*-derived DNA fragment (*EC1*, see Materials and methods) served as positive and negative controls, respectively. From among the 33 candidates, we found a cell wall-associated kinase (WAK) gene (*SlWAK1*, Solyc09g014720), whose silencing compromised PTI-associated cell death suppression (Figure 5A). *SlWAK1*-silenced plants were morphologically similar to *FLS2* and *EC1* silencing controls (Additional file 7: Figure S4).

To examine the effect of *SlWAK1* on immunity further, we initially performed disease assays using either vacuum infiltration or dip inoculation of the *N. benthamiana* pathogen DC3000Δ*hopQ1-1* at 5 × 10^4^ cfu/mL but we did not see any bacterial growth or disease symptom differences among *EC1*, *FLS2-* or *SlWAK1-*silenced plants. As an alternative, we first infiltrated plants with *P. fluorescens* at 2 × 10^7^ cfu/mL to induce PTI and 6 h later infiltrated the plants with DC3000Δ*hopQ1-1* at 10^5^ cfu/mL. Using these conditions, we detected reproducible and statistically significant (*P* < 0.01) higher bacterial growth in plants silenced for *SlWAK1* compared with the *EC1* controls (Figure 5B). In *FLS2*-silenced plants bacterial growth was intermediate between that of *EC1* and *SlWAK1* plants, particularly at 3 days after inoculation (dai), but not statistically different from *EC1* control plants. Disease symptoms were initially observed at 2 dai, and became more apparent at 7 dai (Figure 5C). *SlWAK1-*silenced plants had leaf necrosis similar to *FLS2*-silenced plants and both had more disease symptoms compared to *EC1* control plants.

**Figure 6:**
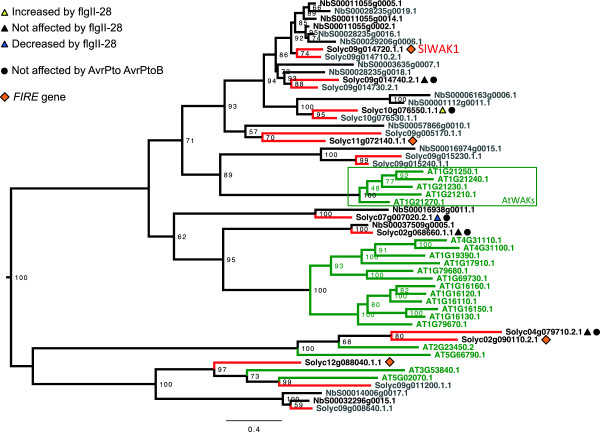
**Phylogenetic analysis of WAK and WAKL amino acid sequences from tomato, *Arabidopsis *and *Nicotiana benthamiana.*** Green font and lines indicate *Arabidopsis* proteins. Black and red lines indicate *N. benthamiana* and tomato, respectively. The light gray accession numbers for tomato and *N. benthamiana* are proteins for whose genes few or no RPKM were detected. For all comparisons a ≥2-fold difference and P < 0.05 were used as cut-offs. RPKM, reads per kilobase of exon model per million mapped reads.

There are 7 *SlWAK* and 16 *SlWAK-Like (SlWAKL)* predicted genes in tomato [30]. *SlWAK1* is a part of a clustered four-gene family; two of these are not expressed in our conditions (Solyc09g014710 and Solyc09g014730) and expression of the other was constitutive (Solyc09g014740). A phylogenetic analysis of the WAK and WAKL proteins from *Arabidopsis*, *N. benthamiana* and tomato (Figure 6) showed that, as expected, those from the solanaceous species cluster together. Although some orthologous relationships may be evident, the solanaceous WAKs and WAKLs are generally distinct from those in *Arabidopsis.* Interestingly, Solyc09g015230 and Solyc09g015240, the tomato *WAKs* that are most closely related to the well-studied *Arabidopsis WAK* genes [31], are not expressed in tomato leaves. SlWAK1 clusters with several *N. benthamiana* and tomato proteins distinct from the *Arabidopsis* WAK proteins, suggesting the WAKs in these solanaceous species do not have a clear orthology to any of the *Arabidopsis* WAKs.

We took advantage of the draft genome sequence of *N. benthamiana*[32] and a phylogenetic analysis based on nucleotide sequences to identify putative orthologs and possible off-targets of the *SlWAK1* VIGS fragment using stringent conditions (that is, genes with ≥17 nucleotides of 100% identity). Additionally, we generated an RNA-seq dataset from *N. benthamiana* leaves infiltrated with *P. fluorescens* 55 at 10^8^ cfu/mL, which we used to remove genes with low or no detectable transcripts from this analysis (< 3 RPKM; Additional file 8: Table S4). This approach showed that our VIGS construct was predicted to silence three closely related *WAK* genes in *N. benthamiana* (Additional file 9: Table S5). Quantification of the transcript abundance of these genes by quantitative reverse transcription-PCR (qRT-PCR) supported this prediction as NbS00011055g0005, NbS00011055g0014 and NbS00011055g0002 each had decreased transcript abundance, ranging between 45% and 65%, in plants silenced using *SlWAK1* (Figure 5D). We chose one of the closest expressed predicted non-target genes (NbS00016938g0011, Additional file 10: Figure S5) and saw no difference in transcript abundance in *SlWAK1*-silenced plants (Figure 5D). Thus the increased susceptibility to *Pst* that we observe in *SlWAK1*-silenced plants may be due to either one or a combination of three *SlWAK1* orthologs in *N. benthamiana* but is unlikely to be due to off-target silencing.

## Discussion

We observed that about 13% of the genes expressed in leaves under our conditions were induced 6 h after exposure to a single flagellin-derived MAMP. We hypothesized that *Pseudomonas syringae* pv. *tomato* (*Pst*) has evolved to interfere with many of the most important PTI-induced genes and so further focused on those genes whose expression is suppressed by two pathogen effector proteins, AvrPto and AvrPtoB. These effectors target PRR complexes as part of their virulence activity and are expected to compromise a large portion of the PTI response emanating from subsequent signaling events [14]. Of the 2,268 flgII-28-induced genes, 27% were present in this *flagellin-induced, repressed by effector* (*FIRE*) class. At least 25 *FIRE* genes have been previously implicated in the plant immune response including *Bti9* (Solyc02g079600), *FLS2* (Solyc02g070890 and Solyc02g070910), *RIN4* (Solyc09g059430 and Solyc06g083390), *HvCRK1* (Solyc03g111540) and *LCERK-VI.2* (Solyc09g005000) (Additional file 5: Table S2). From an initial screen of 33 protein kinase-encoding *FIRE* genes, we identified *SlWAK1*, a gene that, when silenced, compromised the host immune response to *Pst* infection to a similar degree as that observed in *FLS2*-silenced plants. Together, the collection of 622 *FIRE* genes provides a new resource for functional genomics screens in solanaceous plants and for comparative transcriptomics of PTI and ETI responses induced by specific MAMPs and DAMPs and diverse pathogens.

Plants recognize several bacterial MAMPs including flg22, EF-Tu, csp22, peptidoglycan and lipopolysaccharides, although the importance of each of these in any particular plant species varies [4]. Recently, a new flagellin-derived MAMP, flgII-28, has been discovered that is recognized by tomato and other solanaceous plants but not by *Arabidopsis*[12]. The possibility that FLS2 might be involved in flgII-28 recognition has been excluded and its receptor remains unknown [13]. Here we used flgII-28 to elicit PTI in tomato leaves and found that it induces large transcriptional changes similar to host responses to other MAMPs. These changes largely overlap with those observed when the FliC protein is used to elicit PTI as shown by comparisons of wild-type and flagellin-deficient bacteria. Together, our RNA-seq experiments indicate that flagellin-derived MAMPs (flg22 and/or flgII-28) are by the far the major bacterial MAMP recognized by tomato 6 h post inoculation. We cannot exclude the possibility that other MAMPs play an important role at other time points, in specific plant tissues or at certain stages of pathogen attack. For example, we have reported that the Bti9 kinase (the AtCERK1 ortholog in tomato) likely detects a flagellin-independent MAMP and makes an important contribution to *Pst* resistance, especially in the lower leaves of tomato [16]. It also has been discovered that in *Arabidopsis* seedlings the transcriptional response to oligogalacturonides (OGs) and flg22 greatly overlaps 1 h after exposure, but at 3 h the flg22 transcriptional response is still high while the response to OGs is diminished, suggesting that transcriptional reprogramming due to perception of these two elicitors changes over time [23]. Further experiments with additional MAMPs and at multiple time points will be needed to understand better possible temporal effects on the host response to bacterial elicitors.

The central importance of flagellin detection in the activation of the PTI response in tomato makes it a vulnerable target for bacterial pathogens. Currently, five *Pst* effectors are known to undermine the FLS2-BAK1 pathway (AvrPto, AvrPtoB, AvrPphB, HopAI1 and HopF2) [6]. Examination of a ‘minimal repertoire’ of type III effectors needed to confer full virulence to *Pst* strain DC3000 revealed that AvrPto and AvrPtoB are ‘early acting’ effectors whose activity allows for the manipulation by later acting effectors of other host defenses such as vesicle trafficking [5]. This early activity is due to the fact that AvrPto and AvrPtoB each have a domain that binds and inhibits the kinase domains of proteins in the FLS2/BAK1 complex [14]. Our finding that about 30% of the genes induced by flgII-28 are suppressed by AvrPto and/or AvrPtoB suggests they may also disrupt the flgII-28 receptor complex. Each of these effectors also has other domains with virulence-promoting activities [15,16,33-35]. Whether these domains affect host gene expression is unknown, but our present RNA-seq data suggest that transcriptomics combined with point mutations in each effector, which affect the virulence determinants independently, could help elucidate the contributions of each domain.

Our initial VIGS screen of 33 kinase-encoding *FIRE* genes identified a plant cell wall-associated kinase (*SlWAK1*) that plays an important role in resistance to infection by *Pst. WAK* genes were first identified in *Arabidopsis* where they occur as a clustered five-member gene family along with 21 other ‘WAK-like kinases’ (*WAKL*) elsewhere in the genome [31,36,37]. WAK and most WAKL proteins contain a cytoplasmic kinase domain, a transmembrane domain and extracellular epidermal growth factor motifs [37]. These proteins have been implicated previously in host responses to pathogen attack and particularly in the perception of pectin-derived fragments [31]. WAKLs were found to be highly represented among the kinases induced by MAMPs in *Arabidopsis* although no specific gene was shown to contribute to the immune response [22]. In fact, a clear demonstration of the role of WAKs in host defense by loss-of-function analyses has been hindered by lethality or developmental defects of some *WAK* mutants and possibly redundancy, which is not easily addressed by a mutational approach, due to the tight linkage of the five *WAK* genes in *Arabidopsis*[38]. As a result, the best evidence to date that WAKs play a role in immunity comes from indirect approaches. For example, overexpression of chimeric receptors using domains of WAK1 and EFR revealed that WAK1 is a receptor for OGs and can contribute to resistance to *Agrobacterium tumefaciens* and *Botrytis cinerea*[39]. In another study, a dominant allele of *WAK2*, which was created by fusing it to an artificial epitope tag, induced various defense-associated responses that were suppressed by a *mpk6* mutation [40]. In addition, certain WAKLs have been implicated in resistance to fungal pathogens [41,42] and recently *AtWAKL10* was reported to be expressed coordinately with defense-related genes [43].

In our case, silencing reduced the transcript abundance of three closely-related *WAK* genes in *N. benthamiana* by 45% to 65% and resulted in severely compromised PTI and enhanced susceptibility to *Pst* infection. There are seven predicted *WAK* genes in tomato and four of them occur in a clustered gene family that includes *SlWAK1*. Unlike genes belonging to the *WAK* family in *Arabidopsis*[23], *SlWAK1* gene expression is induced during the response to MAMPs. Extensive sequence divergence prevented the assignment of orthologous relations between the tomato *WAK* genes and those in *Arabidopsis*. Consequently, we designated our gene as *SlWAK1*, since it appears to be the first *WAK* gene to be described in tomato. The previously reported *LeWAK* (Solyc02g090110) [44], is actually a *WAKL* and is most closely related to *Arabidopsis WAKL14* (AT2G23450) and *WAKL21* (AT5G66790) (Figure 6). *LeWAK* is also a *FIRE* gene and we silenced it along with two other *FIRE WAK/L* genes but observed no effect on PTI.

Based on previous work with *Arabidopsis*, it is possible that SlWAK1 acts as a DAMP receptor by playing a role in recognition of OGs and subsequent signal transduction. OGs could be generated from enzymatic activity encoded by either the pathogen or the host [45]. For example, DC3000 has both a pectin lyase (PL) and a polygalacturonase (PG), which could generate OGs during the infection process [46]. In addition, tomato has a *PG* gene that was shown to be induced in leaves by damage (Solyc08g060970) and, interestingly, this is a *FIRE* gene. Another *FIRE* gene, Solyc05g005570, is closely related to a gene encoding a PG beta subunit that modulates PG activity (Solyc05g005560). Inhibitors of PG activity have been implicated in contributing to the formation of pectin fragments that function as DAMPs [3,45]. A gene encoding one of these inhibitors is induced by flagellin, but is not a *FIRE* gene (Solyc07g065090). Collectively, our observations support a model in which tomato recognition of MAMPs leads to increased expression of *SlWAK1* and an increase in SlWAK1 receptors at the plasma membrane. Subsequently, OGs are generated by pathogen- and/or host-encoded enzymes and are recognized as DAMPs by SlWAK1, triggering a sustained immune response. Further experiments are needed to test this model, such as assaying whether SlWAK1 participates in OG perception, developing and testing DC3000 mutants that lack the *PL* and/or *PG* genes, and examining the effect of silencing the host *PG* and *PG* beta subunit genes.

Forward and reverse genetic screens for genes that act downstream of MAMP recognition have identified relatively few genes [2,29]. Instead, downstream components have mostly been discovered by biochemical (BAK1) or transcriptomic (BIK1) approaches [47,48]. This apparent shortcoming of genetic screens may be due to functional redundancy of specific genes or of PTI-induced pathways. It is also possible that, as with BAK1, many PTI-related genes play an important role in development or other fundamental processes and these genes might be missed by forward genetic screens. Here, we excluded from our VIGS screen those genes that have already been shown to be involved in immunity (25 out of 622). The fact that just one gene in the first 33 tested genes had a compromised immune response might indicate that relatively few PTI-induced genes play a critical role in immunity. Alternatively, this low frequency could be due to the use of heterologous fragments for VIGS silencing, limitations of the one PTI assay we used or redundancy in gene function. Consistent with the last possibility, multiple calcium-dependent protein kinases had to be knocked out to observe their involvement in immunity [49]. An initial challenge for such combinatorial genetic approaches to functional testing of redundant factors is the identification of a set of candidate genes that is small enough to be manageable and sufficiently documented to justify the effort. The *FIRE* genes we have identified here meet these criteria. Furthermore, our transcriptomic and phenotypic analyses of *FIRE* genes suggest that tomato defense against *Pst* is dominated by perception of a single MAMP and one or more DAMPs. Importantly, the *FIRE* gene set provides a new tool for unraveling the redundancies in downstream kinase signaling that are predicted to confer system robustness in the face of pathogen effector attack.

## Conclusions

We performed an extensive RNA-seq analysis and VIGS screen to identify novel genes contributing to the plant immune response. The host response to flgII-28 and various bacterial strains indicated that flagellin is by far the major MAMP recognized by tomato. A *Pseudomonas syringae* mutant lacking two early acting effectors (AvrPto and AvrPtoB) allowed the identification of a defined set of *FIRE* genes. Among these *FIRE* genes, 25 have been previously implicated in plant defense highlighting the relevance of this ‘pathogen-defined’ set of genes. A wall-associated kinase was identified, which, when silenced, compromised the plant immune response to bacterial infection. The *FIRE* genes provide a unique resource for dissection of the plant immune system.

## Materials and methods

### Bacterial strains and growth conditions

Bacterial strains used in this work are listed in Additional file 11: Table S6. *Pseudomonas* strains were grown on King’s B medium at 30°C. *Agrobacterium tumefaciens* and *E. coli* were grown on Luria–Bertani (LB) medium at 30°C and 37°C, respectively. Antibiotics used were: ampicillin (100 μg/mL), kanamycin (50 μg/mL), rifampicin (10 μg/mL) and spectinomycin (50 μg/mL).

### Plant material and treatments

Plants were grown at 75% humidity with 16 h light (day 24°C, night 22°C). Four leaflets of the third true leaf of 4-week-old Rio Grande *prf3* tomato plants were syringe-infiltrated and kept at 24°C and 75% humidity during the experiment. Infiltrations consisted of 1 μM flgII-28 (ESTNILQRMRELAVQSRNDSNSSTDRDA, EZBiolab; 90% purity) or bacterial strains (Table 1). *Nicotiana benthamiana* leaves were vacuum-infiltrated with *Pseudomonas fluorescens* 55 (*P. fluorescens*) at 10^8^ cfu/mL and sampled 6 h later. Mock-treated and untreated tissues were also collected. Three biological replicates (successive weekly treatments) were performed for each treatment. Tissue was collected at 6 h after infiltration, immediately frozen in liquid N_2_ and stored at -80°C until used.

### RNA sequencing analysis

Total RNA was isolated using TRIzol reagent (Life Technologies, Grand Island, NY, USA) and libraries for sequencing were constructed as described [50]. Barcoded libraries were multiplexed by eight in each lane and sequenced on an Illumina Hiseq 2000 system using the single-end mode. The length of the reads was 45 or 50 bp. Sequence reads have been deposited in the National Center for Biotechnology Information sequence read archive under accession number SRA096750. RNA-seq reads were first aligned to a ribosomal RNA sequence database [51] using Bowtie [52] and the aligned read sequences were removed. The remaining reads were aligned to the tomato genome sequence (version 2.40) using TopHat [53]. Detailed information on the quality of reads in each replicate is provided in Additional file 12: Table S7. Following alignment, for each gene model, the count of mapped reads from each sample was derived and normalized to RPKM (reads per kilobase of exon model per million mapped reads). Differentially expressed genes were identified using the DESeq 1.8.3 package [54] with the raw count data. Raw *P* values were corrected for multiple testing using the false discovery rate [55].

For 20% of the genes, we detected 0 RPKM indicating these are probably not expressed (or expressed at very low levels) in overall leaf tissue in our experimental conditions. In a eukaryotic cell with 200,000 mRNA molecules, 5 RPKM represents one detected mRNA per cell. Leaf tissue is a mixture of different cell populations, and the RPKM of a highly expressed but cell-specific gene could be masked by the scarcity of the cell type. Hence, there is no consensus on how to select and remove from the analysis poorly expressed genes, although cut-offs ranging from 0.3 to 10 RPKM are found in the literature [56,57]. In our case, to set a meaningful RPKM level, we examined the RPKM of a subset of genes known to play a role in plant immunity, including *FLS2* (AT5G046330; Solyc02g070890 and Solyc02g070910), *Rin4* (AT3G25070, Solyc06g083390 and Solyc09g059430), *SlSERK3A* (AT4G33430, Solyc10g047140) and *Bti9* (Solyc02g079600). Of these genes, *FLS2.2* (Solyc02g070910) had the lowest values ranging from 0.12 to 6.45 RPKM with 3 RPKM in the *P. fluorescens* treatment (Additional file 1: Figure S1). Based on this information we included in our analysis genes with ≥3 RPKM in at least one treatment (approximately 39 reads mapped per kilobase of transcript for an average of 13 million mapped reads; Additional file 12: Table S7).

### Virus-induced gene silencing

For silencing, inserts of 300 to 400 bp were chosen based on a BLAST analysis of the *N. benthamiana* genome sequence [58]. Primers were designed using Primer3 [59]. PCR amplification was performed using cDNA from Rio Grande *prf3* tomato leaves treated with flgII-28 (1 μM for 6 h; EZ Biolabs, Carmel, IN, USA) or *N. benthamiana* leaves treated with flg22 (1 μM for 6 h, GenScript, Piscataway, NJ, USA). PCR products were cloned into pCR8/GW/TOPO (Life Technologies) and recombined into a Gateway-compatible TRV2 vector [60]. After sequence confirmation, constructs were transformed into *A. tumefaciens* GV2260. VIGS in *N. benthamiana* plants was performed as described [61]. An *FLS2* fragment and an *E. coli* gene-based fragment (*EC1*, which contains a 56% GC content and not a single ≥17 bp-long 100% identical stretch in *N. benthamiana*) were used as positive and negative controls. The DNA sequence of the fragments used were:

#### >EC1 (Escherichia *coli* fragment)

CGGCGTGATTGCGCAAAGCTATCATCAGTCTGAGAAATCGGCCTCCGAGTTCGATGCCATTGTTGCGCAAACGGAGCAGTTCCTTGCCGACAATGGTCGTCGCCCGCGCATTCTGATCGCTAAGATGGGCCAGGATGGACACGATCGCGGCGCGAAAGTGATCGCCAGCGCCTATTCCGATCTCGGTTTCGACGTAGATTTAAGCCCGATGTTCTCTACACCTGAAGAGATCGCCCGCCTGGCCGTAGAAAACGACGTTCACGTAGTGGGCGCATCCTCACTGGCTGCCGGTCATAAAACGCTGATCCCGGAACTGGTCGAAGCGCTGAAAAAATGGGGACGCGAAGATATCTGCGTGGTCGCGGGTGGCGTCATTCCGCCGCAGGATTACGCCTTCCTGCAAGAGCGCGGCGTGGCGGCGATTTATGGTCCAGGTACACCTATGCTCGACAGTGTGCGCGACGTACTGAATCTGATAAGCCAGCATCATGATTAATGAAGCCACGCTGGCAGAAAGTATTCG

#### >Tomato *FLS2.1* (Solyc02g070890)

AAAGTGTACCGCAGCACTGAGCCTCCAGAGATTTTATCAAAAGGATTTGGAACATGCTACCAATAATTTCCGTCCGGAAAACATTATTGGAGCCAGCAGTTTAAGTACTGTGTACAAAGGAACACTGGAAGATGGGAAGATTGTAGCAGTTAAGAAGCTGAATCACCAGTTCTCAGCAGAATCTGGTAAATGTTTTGATAGGGAAGTCAAGACTCTGAGCCAACTCAGACACAGGAACCTAGTTAAGGTGCTAGGTTACGCTTGGGAAAGCAAGAAGCTAAGGGCTTTAGTTTTAGAATACATGGAGAATGGGAACTTGGACAACATGATTTATGGTCAAGTAGAGGATGACTGGACGTTGTCCAACAGGATTGATATTTTAGTTTCAGTTGCAAGTGGACTATCATACCTGCATTCAGGCTATGATTTTCCAATAGTGCACTGTGACATGAAGCCTTCAAACATTCTTCTGGACAAAAATATGGAAGCACATGTGAGTGACTTTGGGACGGCTAGGATGTTGGGTATTCA

#### >*SlWAK1* (Solyc09g014720)

AGGCTACAAACAACTATGCCAGTGATAGAATTCTTGGTCGTGGTGGAAATGGAATTGTCTACAAAGGCATTCTATCTGATAATCGCATAGTTGCTATTAAGAAATCTAAGTTTATGGACGAGGAACAGGTTGAACAGTTCATTAACGAGGTACTTATTCTTACTCAAGTCAACCATAGAAATGTTGTGAGACTCTTCGGATGTTGTTTGGAAGCCGAAGTTCCTTTACTTGTCTATGAATACATTTCTCATGGAACTCTTTACGAGCATATCCACAATCGAAATGGAGCACCTTGGTTATCTTGGGAAAATCGGCTAAGAGTTGCTAGTGAGACAGCAAGTGCACTTGCTTACCTTCATTCATCCGCGCAAATGCCTATAATTCATAGAGATGTCAAGTCTGCCAATTTATTGTTGGA

### Cell death suppression assay (pattern-triggered immunity assay)

VIGS plants were tested for compromised PTI as described [29], with minor modifications. Leaves were induced by syringe infiltration with *P. fluorescens* 55 at OD_600_ = 0.5 and 8 h later challenged, in a partially overlapping region, with DC3000 at OD_600_ = 0.01. The appearance of cell death (CD) in the overlapping area was scored at day 7, using the categories: PTI breakdown (>50% of overlapping area with CD), and no PTI breakdown (<50%). In each experiment, four plants per construct were used with eight overlapping infiltrations each. Each experiment was repeated at least three times. Significant differences are based on Dunnett’s method, using TRV:*EC1* plants as controls (*P* < 0.01).

### Bacterial growth assays

Seven-week-old silenced plants were vacuum-infiltrated with a suspension of *P. fluorescens* 55 (2 × 10^7^ cfu/mL) in 10 mM MgCl_2_ and 0.002% Silwet L-77 (Comptom Co). Plants were kept at 24°C and 75% humidity for 6 h and then vacuum-infiltrated with DC3000Δ*hopQ1-1* (10^5^ cfu/mL) suspended in the same solution. To measure bacterial populations, three 0.43 cm^2^ disks were taken from the oldest expanding leaves and processed twice in a Tissue Lyser (Qiagen) for 30 sec at 25/sec frequency with 0.25 mL of 10 mM MgCl_2_. The volume was adjusted to 1 mL and serial dilutions were plated on solid LB medium with antibiotics. In each experiment, six biological replicates per construct were used. Significant differences are based on Dunnett’s method, using TRV:*EC1* plants as controls (*P* < 0.01).

### Gene and treatment clustering and gene ontology term analysis

Genes with an expression value of ≥3 RPKM in at least one treatment and a maximum/minimum ratio ≥3 across all treatments were used for clustering. Hierarchical clustering was performed using Cluster 3.0 [62] with the average linkage clustering method and the Pearson correlation as the measure of similarity. GO term enrichment analysis was based on the GO::TermFinder module [63].

### Phylogenetic analysis and degree of overlap between bacterial strains and flgII-28 treatment

A substitution model was evaluated using JModelTest [64,65] for nucleotide sequences and ProtTest [66] for proteins. PhyML through SeaView [67] was used to perform the analysis with the default parameters for the GTR model (nucleotides) and the JTT model (proteins). One hundred bootstraps were used for each analysis. A tree figure was created using FigTree [68]. Venny [69] was used to generate Venn diagrams to study the degree of overlap between genes affected by flgII-28 and different bacterial strains treatments.

### Identification of *FIRE* immunity-associated genes

A bibliographic search associated with the *FIRE* genes was performed through: (1) a SQL search of the Sol Genomic Network database [70] using the Solyc identifiers in the Phenome and Chado schema that contains the manually curated loci and (2) a Swissprot database search of the *Arabidopsi*s homologous genes (identified using the best match of a Selfblast search). The Swissprot database was mined using a Perl script (available upon request).

### Virus-induced gene silencing efficiency

Six *N. benthamiana* silenced plants per construct were vacuum-infiltrated with *P. fluorescens* 55 as described above and samples taken at 5, 6, 7 and 8 h later. Tissues from the same plant were pooled and the resulting RNA used for quantitative reverse transcription-PCR (qRT-PCR). Total RNA was isolated using the Plant RNA isolation reagent (Life Technologies, Grand Island, NY, USA) according to the manufacturer’s instructions. Then 6 μg of total RNA was processed with the TURBO DNA-free kit (Life Technologies) for 60 min at 37°C. After DNase treatment, 2 μg RNA was used to prepare cDNA using the SuperScript III First-Strand Synthesis System (Life Technologies) with oligo(dT)20. qRT-PCR was performed as described previously [71]. The sequence of the primers used for each gene analyzed were: NbS00011055g0005.1 F 5′-GAAATATCCCACGGTGATCC-3′, NbS00011055g0005.1R 5′-GAAGAATGACTGCGGTTAGG-3′; NbS00011055g0014.1 F 5′-CCCGTTACTCAATACGTTCTT-3′, NbS00011055g0014.1R 5′-ATTGGGCGGTGGTTAATG-3′; NbS00011055g0002.1 F 5′-AACAATATCCCACGGTGAC-3′ and NbS00011055g0002.1R 5′-TTAAAGGAAGACGCGAAGG-3′. Cycling conditions during qRT-PCR were 50°C for 2 min, 95°C for 10 min, and 40 cycles of 95°C for 30 s, 55°C for 30 s and 72°C for 30 s. Data were normalized to the *NbPP2a* and *NbEF1α* genes [72]. The significance of the expression data was analyzed using a pairwise Student’s *t*-test (*P* < 0.01).

## Abbreviations

bp: Base pair; CD: Cell death; CDSA: Cell death suppression assay; dai: Days after inoculation; DAMP: Damage-associated molecular pattern; ETI: Effector-triggered immunity; FIRE: Flagellin-induced repressed by effectors; GO: Gene ontology; LRR: Leucine-rich repeat; MAMP: Microbe-associated molecular pattern; OG: Oligogalacturonides; PCR: Polymerase chain reaction; PG: Polygalacturonase; PL: Pectin lyase; PRR: Pattern recognition receptor; Pst: *Pseudomonas syringae* pv. *tomato*; PTI: Pattern-triggered immunity; qRT-PCR: Quantitative reverse transcription-PCR; RNA-seq: RNA sequencing; RPKM: Reads per kilobase of exon model per million mapped reads; TF: Transcription factor; VIGS: Virus-induced gene silencing; WAK: Wall-associated kinase.

## Competing interests

The authors declare that they have no competing interests.

## Authors’ contributions

HGR, ZF, AC and GBM designed the research. SZ contributed new reagents and analytic tools. HGR and MAP performed the research. HGR, YZ, MAP, AB, ZF and GBM analyzed the data. HGR and GBM wrote the paper. All authors read and approved the final manuscript.

## Supplementary Material

Additional file 1: Figure S1 Expression level of selected immunity-related genes used for establishing a minimum RPKM cut-off. Bars represent the average of three biological replicates with the corresponding standard error. Arrows highlight transcript abundance differences between wild type DC3000 and DC3000Δ*avrPto*Δ*avrPtoB. FLS2.1* Solyc02g070890, *FLS2.2* Solyc02g070910, *Bti9* Solyc02g079600, *SERK3A* Solyc10g047140, *RIN4.1* Solyc09g059430 and *RIN4.2* Solyc06g083390. Click here for file

Additional file 2: Figure S2 Amino acid sequences of the flgII-28 region in various bacterial strains. Asterisks indicate key residues shown to be important for the elicitation of reactive oxygen species production. Click here for file

Additional file 3: Figure S3 Overlap between genes suppressed by bacterial strains and flgII-28 treatments. **(A)** Overlap between *Pseudomonas syringae* pv. *tomato* strains and flgII-28. **(B)** Overlap between soil *Pseudomonas* and flgII-28. **(C)** Degree of overlap shown as percentage of genes. Click here for file

Additional file 4: Table S1 GO term analysis of genes belonging to clusters A and B (Figure 3). Terms are grouped based on process (P), component (C) and function (F). Click here for file

Additional file 5: Table S2 (A) Extracted *FIRE* gene expression data. (B) Additional immune-related *FIRE* genes identified using less stringent cut-offs. Comment and reference columns show information about genes previously shown to participate in plant immunity based on publications involving tomato and *Arabidopsis* (See Materials and methods for detailed information). Click here for file

Additional file 6: Table S3 GO term analysis of *FIRE* genes using genes induced by flgII-28 as the reference. Terms are grouped based on process (P), component (C) and function (F). Click here for file

Additional file 7: Figure S4 The growth and development of *SlWAK1*-silenced plants are not affected. Click here for file

Additional file 8: Table S4 *WAK/L* RNA-seq gene expression analysis of *N. benthamiana* leaves sampled 6 h post vacuum infiltration with *P. fluorescens* 55. Data shown correspond to the average of three biological replicates per treatment. See Materials and methods for details. Click here for file

Additional file 9: Table S5 *SlWAK1* VIGS construct target analysis in *N. benthamiana*. The length of 100% match regions ≥17 nucleotides long was determined by considering genes with ≥3 RPKM in at least one condition,. Predicted non-target gene information is shown at the bottom of the Table. N.C., not considered due to low or no expression. Click here for file

Additional file 10: Figure S5 Phylogenetic analysis of *WAK* and *WAKL* nucleotide sequences from *N. benthamiana* including *SlWAK1*, which was used to identify putative VIGS construct targets and off-targets. The PhyML method with a bootstrap of 100 replicates was used for the analysis. The bold black font indicates expressed genes with ≥3 RPKM after either mock or *P. fluorescens* (10^8^ cfu/mL) treatment. Color-coded squares show the effect of *P. fluorescens* infiltration using a ≥2-fold difference and *P* < 0.05 as cut-offs. The genes considered to be possible targets of the *SlWAK1* VIGS construct are in clusters A and B and are further described in Additional file 9: Table S5. The asterisk indicates the predicted VIGS non-target gene tested by qRT-PCR (Figure 5). Click here for file

Additional file 11: Table S6 Details of the bacterial strains used in this study. Click here for file

Additional file 12: Table S7 Summary of the sequencing data for each of the libraries generated in this work. Click here for file

## References

[B1] Dodds PN Rathjen JP Plant immunity: towards an integrated view of plant-–pathogen interactions Nat Rev Genet 2010 14 539 548 20585331 10.1038/nrg2812

[B2] Schwessinger B Ronald PC Plant innate immunity: perception of conserved microbial signatures Annu Rev Plant Biol 2012 14 451 482 10.1146/annurev-arplant-042811-105518 22404464

[B3] Nuhse TS Cell wall integrity signaling and innate immunity in plants Front Plant Sci 2012 14 280 23248636 10.3389/fpls.2012.00280 PMC3518785

[B4] Segonzac C Zipfel C Activation of plant pattern-recognition receptors by bacteria Curr Opin Microbiol 2011 14 54 61 10.1016/j.mib.2010.12.005 21215683

[B5] Lindeberg M Cunnac S Collmer A *Pseudomonas syringae* type III effector repertoires: last words in endless arguments Trends Microbiol 2012 14 199 208 10.1016/j.tim.2012.01.003 22341410

[B6] Xin XF He SY *Pseudomonas syringae* pv. *tomato* DC3000: a model pathogen for probing disease susceptibility and hormone signaling in plants Annu Rev Phytopathol 2013 14 473 498 10.1146/annurev-phyto-082712-102321 23725467

[B7] Melotto M Underwood W He SY Role of stomata in plant innate immunity and foliar bacterial diseases Annu Rev Phytopathol 2008 14 101 122 10.1146/annurev.phyto.121107.104959 18422426 PMC2613263

[B8] Gomez-Gomez L Boller T FLS2: an LRR receptor-like kinase involved in the perception of the bacterial elicitor flagellin in *Arabidopsis* Mol Cell 2000 14 1003 1011 10.1016/S1097-2765(00)80265-8 10911994

[B9] Chinchilla D Zipfel C Robatzek S Kemmerling B Nurnberger T Jones JD Felix G Boller T A flagellin-induced complex of the receptor FLS2 and BAK1 initiates plant defence Nature 2007 14 497 500 10.1038/nature05999 17625569

[B10] Zhang J Li W Xiang T Liu Z Laluk K Ding X Zou Y Gao M Zhang X Chen S Mengiste T Zhang Y Zhou JM Receptor-like cytoplasmic kinases integrate signaling from multiple plant immune receptors and are targeted by a *Pseudomonas syringae* effector Cell Host Microbe 2010 14 290 301 10.1016/j.chom.2010.03.007 20413097

[B11] Lu D Wu S Gao X Zhang Y Shan L He P A receptor-like cytoplasmic kinase, BIK1, associates with a flagellin receptor complex to initiate plant innate immunity Proc Natl Acad Sci USA 2010 14 496 501 10.1073/pnas.0909705107 20018686 PMC2806711

[B12] Cai R Lewis J Yan S Liu H Clarke CR Campanile F Almeida NF Studholme DJ Lindeberg M Schneider D Zaccardelli M Setubal JC Morales-Lizcano NP Bernal A Coaker G Baker C Bender CL Leman S Vinatzer BA The plant pathogen Pseudomonas syringae pv. tomato is genetically monomorphic and under strong selection to evade tomato immunity PLoS Pathog 2011 14 e1002130 10.1371/journal.ppat.1002130 21901088 PMC3161960

[B13] Clarke CR Chinchilla D Hind SR Taguchi F Miki R Ichinose Y Martin GB Leman S Felix G Vinatzer BA Allelic variation in two distinct *Pseudomonas syringae* flagellin epitopes modulates the strength of plant immune responses but not bacterial motility New Phytol 2013 14 847 860 10.1111/nph.12408 23865782 PMC3797164

[B14] Martin GB Martin F, Kamoun S Suppression and activation of the plant immune system by *Pseudomonas syringae* effectors AvrPto and AvrPtoB Effectors in Plant-Microbe Interactions 2012 London: Wiley-Blackwell, John Wiley and Sons 123 154

[B15] Yeam I Nguyen HP Martin GB Phosphorylation of the *Pseudomonas syringae* effector AvrPto is required for FLS2/BAK1-independent virulence activity and recognition by tobacco Plant J 2010 14 16 24 10.1111/j.1365-313X.2009.04028.x 19793077

[B16] Zeng L Velasquez AC Munkvold KR Zhang J Martin GB A tomato LysM receptor-like kinase promotes immunity and its kinase activity is inhibited by AvrPtoB Plant J 2012 14 92 103 10.1111/j.1365-313X.2011.04773.x 21880077 PMC3240704

[B17] Pedley KF Martin GB Molecular basis of Pto-mediated resistance to bacterial speck disease in tomato Annu Rev Phytopathol 2003 14 215 243 10.1146/annurev.phyto.41.121602.143032 14527329

[B18] Zhao Y Thilmony R Bender CL Schaller A He SY Howe GA Virulence systems of *Pseudomonas syringae* pv. *tomato* promote bacterial speck disease in tomato by targeting the jasmonate signaling pathway Plant J 2003 14 485 499 10.1046/j.1365-313X.2003.01895.x 14617079

[B19] Zipfel C Robatzek S Navarro L Oakeley EJ Jones JDG Felix G Boller T Bacterial disease resistance in *Arabidopsis* through flagellin perception Nature 2004 14 764 767 10.1038/nature02485 15085136

[B20] Navarro L Zipfel C Rowland O Keller I Robatzek S Boller T Jones JD The transcriptional innate immune response to flg22. Interplay and overlap with Avr gene-dependent defense responses and bacterial pathogenesis Plant Physiol 2004 14 1113 1128 10.1104/pp.103.036749 15181213 PMC514144

[B21] Truman W de Zabala MT Grant M Type III effectors orchestrate a complex interplay between transcriptional networks to modify basal defence responses during pathogenesis and resistance Plant J 2006 14 14 33 10.1111/j.1365-313X.2006.02672.x 16553893

[B22] Thilmony R Underwood W He SY Genome-wide transcriptional analysis of the *Arabidopsis thaliana* interaction with the plant pathogen *Pseudomonas syringae* pv. *tomato* DC3000 and the human pathogen *Escherichia coli* O157:H7 Plant J 2006 14 34 53 10.1111/j.1365-313X.2006.02725.x 16553894

[B23] Denoux C Galletti R Mammarella N Gopalan S Werck D De Lorenzo G Ferrari S Ausubel FM Dewdney J Activation of defense response pathways by OGs and Flg22 elicitors in *Arabidopsis* seedlings Mol Plant 2008 14 423 445 10.1093/mp/ssn019 19825551 PMC2954645

[B24] Mysore KS Crasta OR Tuori RP Folkerts O Swirsky PB Martin GB Comprehensive transcript profiling of Pto- and Prf-mediated host defense responses to infection by *Pseudomonas syringae* pv. *tomato* Plant J 2002 14 299 315 10.1046/j.1365-313X.2002.01424.x 12410809

[B25] de Torres M Sanchez P Fernandez-Delmond I Grant M Expression profiling of the host response to bacterial infection: the transition from basal to induced defence responses in RPM1-mediated resistance Plant J 2003 14 665 676 10.1046/j.1365-313X.2003.01653.x 12609040

[B26] Tomato Genome Consortium The tomato genome sequence provides insights into fleshy fruit evolution Nature 2012 14 635 641 10.1038/nature11119 22660326 PMC3378239

[B27] Cunnac S Chakravarthy S Kvitko BH Russell AB Martin GB Collmer A Genetic disassembly and combinatorial reassembly identify a minimal functional repertoire of type III effectors in *Pseudomonas syringae* Proc Natl Acad Sci USA 2011 14 2975 2980 10.1073/pnas.1013031108 21282655 PMC3041132

[B28] Tomato functional genomics database http://ted.bti.cornell.edu

[B29] Chakravarthy S Velasquez AC Ekengren SK Collmer A Martin GB Identification of *Nicotiana benthamiana* genes involved in pathogen-associated molecular pattern-triggered immunity Mol Plant Microbe Interact 2010 14 715 726 10.1094/MPMI-23-6-0715 20459311

[B30] iTAK - Plant Transcription factor and Protein Kinase Identifier and Classifier http://bioinfo.bti.cornell.edu/cgi-bin/itak/index.cgi

[B31] Kohorn BD Kohorn SL The cell wall-associated kinases, WAKs, as pectin receptors Front Plant Sci 2012 14 88 22639672 10.3389/fpls.2012.00088 PMC3355716

[B32] Bombarely A Rosli HG Vrebalov J Moffett P Mueller LA Martin GB A draft genome sequence of *Nicotiana benthamiana* to enhance molecular plant-microbe biology research Mol Plant Microbe Interact 2012 14 1523 1530 10.1094/MPMI-06-12-0148-TA 22876960

[B33] Xiao F He P Abramovitch RB Dawson JE Nicholson LK Sheen J Martin GB The N-terminal region of *Pseudomonas* type III effector AvrPtoB elicits Pto-dependent immunity and has two distinct virulence determinants Plant J 2007 14 595 614 10.1111/j.1365-313X.2007.03259.x 17764515 PMC2265002

[B34] Nguyen HP Yeam I Angot A Martin GB Two virulence determinants of type III effector AvrPto are functionally conserved in diverse *Pseudomonas syringae* pathovars New Phytol 2010 14 969 982 10.1111/j.1469-8137.2009.03175.x 20122130

[B35] Gimenez-Ibanez S Hann DR Ntoukakis V Petutschnig E Lipka V Rathjen JP AvrPtoB targets the LysM receptor kinase CERK1 to promote bacterial virulence on plants Curr Biol 2009 14 423 429 10.1016/j.cub.2009.01.054 19249211

[B36] He ZH Cheeseman I He D Kohorn BD A cluster of five cell wall-associated receptor kinase genes, *Wak1-5*, are expressed in specific organs of *Arabidopsis* Plant Mol Biol 1999 14 1189 1196 10.1023/A:1006197318246 10380805

[B37] Verica JA He ZH The cell wall-associated kinase (WAK) and WAK-like kinase gene family Plant Physiol 2002 14 455 459 10.1104/pp.011028 12068092 PMC1540232

[B38] De Lorenzo G Brutus A Savatin DV Sicilia F Cervone F Engineering plant resistance by constructing chimeric receptors that recognize damage-associated molecular patterns (DAMPs) FEBS Lett 2011 14 1521 1528 10.1016/j.febslet.2011.04.043 21536040

[B39] Brutus A Sicilia F Macone A Cervone F De Lorenzo G A domain swap approach reveals a role of the plant wall-associated kinase 1 (WAK1) as a receptor of oligogalacturonides Proc Natl Acad Sci USA 2010 14 9452 9457 10.1073/pnas.1000675107 20439716 PMC2889104

[B40] Kohorn BD Kohorn SL Todorova T Baptiste G Stansky K McCullough M A dominant allele of *Arabidopsis* pectin-binding wall-associated kinase induces a stress response suppressed by MPK6 but not MPK3 mutations Mol Plant 2012 14 841 851 10.1093/mp/ssr096 22155845 PMC3399699

[B41] Diener AC Ausubel FM *Resistance to Fusarium oxysporum 1*, a dominant *Arabidopsis* disease-resistance gene, is not race specific Genetics 2005 14 305 321 10.1534/genetics.105.042218 15965251 PMC1456520

[B42] Li H Zhou SY Zhao WS Su SC Peng YL A novel wall-associated receptor-like protein kinase gene, *OsWAK1*, plays important roles in rice blast disease resistance Plant Mol Biol 2009 14 337 346 10.1007/s11103-008-9430-5 19039666

[B43] Meier S Ruzvidzo O Morse M Donaldson L Kwezi L Gehring C The *Arabidopsis wall associated kinase-like 10* gene encodes a functional guanylyl cyclase and is co-expressed with pathogen defense related genes PLoS One 2010 14 e8904 10.1371/journal.pone.0008904 20126659 PMC2811198

[B44] Lejeune A Constant S Delavault P Simier P Thalouarn P Thoiron S Involvement of a putative *Lycopersicon esculentum* wall-associated kinase in the early steps of tomato–*Orobanche ramosa* interaction Physiol Molec Plant Pathol 2006 14 3 12 10.1016/j.pmpp.2006.12.001

[B45] Ferrari S Savatin DV Sicilia F Gramegna G Cervone F Lorenzo GD Oligogalacturonides: plant damage-associated molecular patterns and regulators of growth and development Front Plant Sci 2013 14 49 23493833 10.3389/fpls.2013.00049 PMC3595604

[B46] Buell CR Joardar V Lindeberg M Selengut J Paulsen IT Gwinn ML Dodson RJ Deboy RT Durkin AS Kolonay JF Madupu R Daugherty S Brinkac L Beanan MJ Haft DH Nelson WC Davidsen T Zafar N Zhou L Liu J Yuan Q Khouri H Fedorova N Tran B Russell D Berry K Utterback T Van Aken SE Feldblyum TV D’Ascenzo M The complete genome sequence of the *Arabidopsis* and tomato pathogen *Pseudomonas syringae* pv. *tomato* DC3000 Proc Natl Acad Sci USA 2003 14 10181 10186 10.1073/pnas.1731982100 12928499 PMC193536

[B47] Nam KH Li J BRI1/BAK1, a receptor kinase pair mediating brassinosteroid signaling Cell 2002 14 203 212 10.1016/S0092-8674(02)00814-0 12150928

[B48] Veronese P Nakagami H Bluhm B Abuqamar S Chen X Salmeron J Dietrich RA Hirt H Mengiste T The membrane-anchored Botrytis-induced kinase1 plays distinct roles in *Arabidopsis* resistance to necrotrophic and biotrophic pathogens Plant Cell 2006 14 257 273 10.1105/tpc.105.035576 16339855 PMC1323497

[B49] Boudsocq M Willmann MR McCormack M Lee H Shan L He P Bush J Cheng SH Sheen J Differential innate immune signalling via Ca^2+^ sensor protein kinases Nature 2010 14 418 422 10.1038/nature08794 20164835 PMC2841715

[B50] Zhong S Joung JG Zheng Y Chen YR Liu B Shao Y Xiang JZ Fei Z Giovannoni JJ High-throughput Illumina strand-specific RNA sequencing library preparation Cold Spring Harb Protoc 2011 14 940 949 21807852 10.1101/pdb.prot5652

[B51] Quast C Pruesse E Yilmaz P Gerken J Schweer T Yarza P Peplies J Glockner FO The SILVA ribosomal RNA gene database project: improved data processing and web-based tools Nucleic Acids Res 2013 14 D590 D596 10.1093/nar/gks1219 23193283 PMC3531112

[B52] Langmead B Trapnell C Pop M Salzberg SL Ultrafast and memory-efficient alignment of short DNA sequences to the human genome Genome Biol 2009 14 R25 10.1186/gb-2009-10-3-r25 19261174 PMC2690996

[B53] Trapnell C Pachter L Salzberg SL TopHat: discovering splice junctions with RNA-Seq Bioinformatics 2009 14 1105 1111 10.1093/bioinformatics/btp120 19289445 PMC2672628

[B54] Anders S Huber W Differential expression analysis for sequence count data Genome Biol 2010 14 R106 10.1186/gb-2010-11-10-r106 20979621 PMC3218662

[B55] Benjamini Y Hochberg Y Controlling the false discovery rate: a practical and powerful approach to multiple testing J Royal Stat Soc Series B (Methodological) 1995 14 289 300

[B56] Ramskold D Wang ET Burge CB Sandberg R An abundance of ubiquitously expressed genes revealed by tissue transcriptome sequence data PLoS Comput Biol 2009 14 e1000598 10.1371/journal.pcbi.1000598 20011106 PMC2781110

[B57] Castruita M Casero D Karpowicz SJ Kropat J Vieler A Hsieh SI Yan W Cokus S Loo JA Benning C Pellegrini M Merchant SS Systems biology approach in *Chlamydomonas* reveals connections between copper nutrition and multiple metabolic steps Plant Cell 2011 14 1273 1292 10.1105/tpc.111.084400 21498682 PMC3101551

[B58] SOL genomics network http://solgenomics.net/

[B59] Rozen S Skaletsky H Primer3 on the WWW for general users and for biologist programmers Methods Mol Biol 2000 14 365 386 10547847 10.1385/1-59259-192-2:365

[B60] Liu Y Schiff M Dinesh-Kumar SP Virus-induced gene silencing in tomato Plant J 2002 14 777 786 10.1046/j.1365-313X.2002.01394.x 12220268

[B61] Velasquez AC Chakravarthy S Martin GB Virus-induced gene silencing (VIGS) in *Nicotiana benthamiana* and tomato J Vis Exp 2009 14 e1292 10.3791/1292 PMC2795700 19516240

[B62] de Hoon MJ Imoto S Nolan J Miyano S Open source clustering software Bioinformatics 2004 14 1453 1454 10.1093/bioinformatics/bth078 14871861

[B63] Boyle EI Weng S Gollub J Jin H Botstein D Cherry JM Sherlock G GO::TermFinder – open source software for accessing Gene Ontology information and finding significantly enriched Gene Ontology terms associated with a list of genes Bioinformatics 2004 14 3710 3715 10.1093/bioinformatics/bth456 15297299 PMC3037731

[B64] Darriba D Taboada GL Doallo R Posada D jModelTest 2: more models, new heuristics and parallel computing Nat Methods 2012 14 772 22847109 10.1038/nmeth.2109 PMC4594756

[B65] Guindon S Gascuel O A simple, fast, and accurate algorithm to estimate large phylogenies by maximum likelihood Syst Biol 2003 14 696 704 10.1080/10635150390235520 14530136

[B66] Abascal F Zardoya R Posada D ProtTest: selection of best-fit models of protein evolution Bioinformatics 2005 14 2104 2105 10.1093/bioinformatics/bti263 15647292

[B67] Gouy M Guindon S Gascuel O SeaView version 4: a multiplatform graphical user interface for sequence alignment and phylogenetic tree building Mol Biol Evol 2010 14 221 224 10.1093/molbev/msp259 19854763

[B68] FigTree http://tree.bio.ed.ac.uk/software/figtree/

[B69] Oliveros JC VENNY. An interactive tool for comparing lists with Venn diagrams 2007 http://bioinfogp.cnb.csic.es/tools/venny/

[B70] Bombarely A Menda N Tecle IY Buels RM Strickler S Fischer-York T Pujar A Leto J Gosselin J Mueller LA The Sol Genomics Network (solgenomics.net): growing tomatoes using Perl Nucleic Acids Res 2011 14 D1149 D1155 10.1093/nar/gkq866 20935049 PMC3013765

[B71] Nguyen HP Chakravarthy S Velasquez AC McLane HL Zeng L Nakayashiki H Park DH Collmer A Martin GB Methods to study PAMP-triggered immunity using tomato and *Nicotiana benthamiana* Mol Plant Microbe Interact 2010 14 991 999 10.1094/MPMI-23-8-0991 20615110

[B72] Liu D Shi L Han C Yu J Li D Zhang Y Validation of reference genes for gene expression studies in virus-infected *Nicotiana benthamiana* using quantitative real-time PCR PLoS One 2012 14 e46451 10.1371/journal.pone.0046451 23029521 PMC3460881

