# Supplementary material for: Transcriptomics-based screen for genes induced by flagellin and repressed by pathogen effectors identifies a cell wall-associated kinase involved in plant immunity
Source: Genome Biol. 2013 Dec 20;14(12):R139. doi: 10.1186/gb-2013-14-12-r139 (PMC4053735; doi:10.1186/gb-2013-14-12-r139)
Supplement: Additional file 1: Figure S1 — Expression level of selected immunity-related genes used for establishing a minimum RPKM cut-off. Bars represent the average of three biological replicates with the corresponding standard error. Arrows highlight transcript abundance differences between wild type DC3000 and DC3000ΔavrPtoΔavrPtoB. FLS2.1 Solyc02g070890, FLS2.2 Solyc02g070910, Bti9 Solyc02g079600, SERK3A Solyc10g047140, RIN4.1 Solyc09g059430 and RIN4.2 Solyc06g083390. [file gb-2013-14-12-r139-S1.pdf]

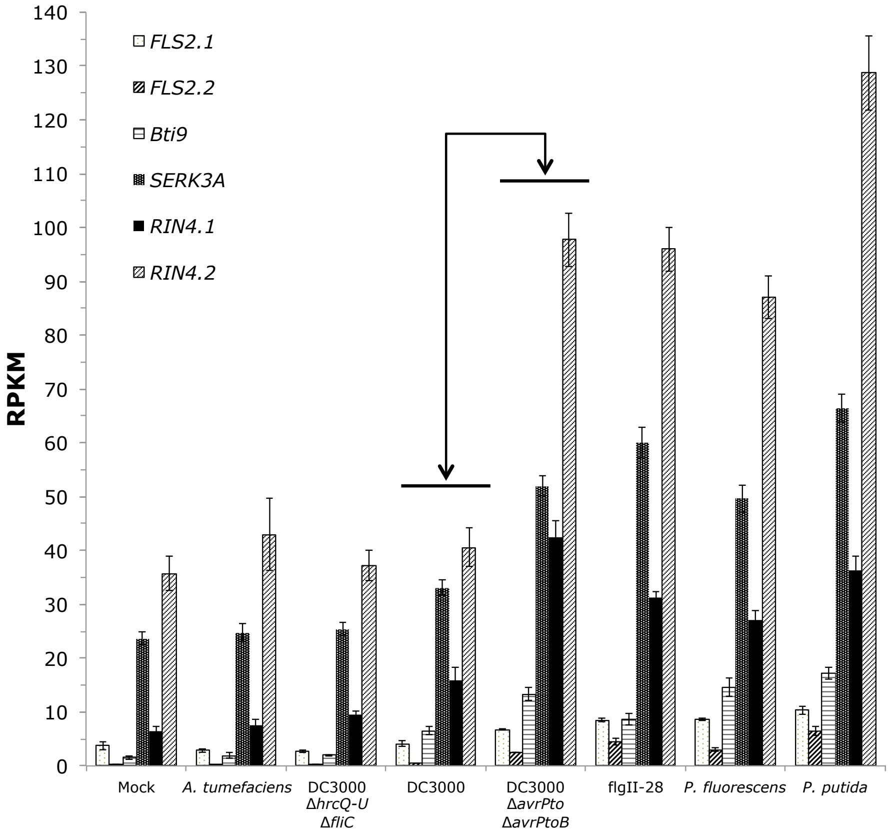

Additional file 1: Figure S1. Expression level of selected immunity-related genes used for establishing a minimum RPKM cut off (see Methods). Bars represent the average of three biological replicates with the corresponding standard error. Arrows highlight transcript abundance differences between wild type DC3000 and DC3000Δ*avrPto*Δ*avrPtoB*. *FLS2.1* Solyc02g070890, *FLS2.2* Solyc02g070910, *Bti9* Solyc02g079600, *SERK3A* Solyc10g047140, *RIN4.1* Solyc09g059430 and *RIN4.2* Solyc06g083390.
