# Supplementary material for: Transcriptomics-based screen for genes induced by flagellin and repressed by pathogen effectors identifies a cell wall-associated kinase involved in plant immunity
Source: Genome Biol. 2013 Dec 20;14(12):R139. doi: 10.1186/gb-2013-14-12-r139 (PMC4053735; doi:10.1186/gb-2013-14-12-r139)
Supplement: Additional file 2: Figure S2 — Amino acid sequences of the flgII-28 region in various bacterial strains. Asterisks indicate key residues shown to be important for the elicitation of reactive oxygen species production. [file gb-2013-14-12-r139-S2.pdf]

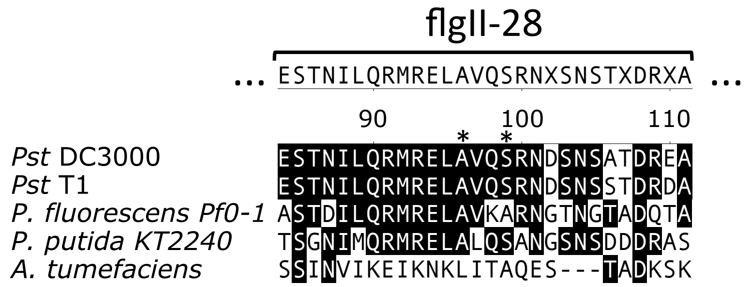

Additional file 2: Figure S2. Amino acid sequences of the flgII-28 region in various bacterial strains. Asterisks indicate key residues shown to be important for the elicitation of reactive oxygen species production [1].
