# Supplementary material for: Transcriptomics-based screen for genes induced by flagellin and repressed by pathogen effectors identifies a cell wall-associated kinase involved in plant immunity
Source: Genome Biol. 2013 Dec 20;14(12):R139. doi: 10.1186/gb-2013-14-12-r139 (PMC4053735; doi:10.1186/gb-2013-14-12-r139)
Supplement: Additional file 3: Figure S3 — Overlap between genes suppressed by bacterial strains and flgII-28 treatments. (A) Overlap between Pseudomonas syringae pv. tomato strains and flgII-28. (B) Overlap between soil Pseudomonas and flgII-28. (C) Degree of overlap shown as percentage of genes. [file gb-2013-14-12-r139-S3.pdf]

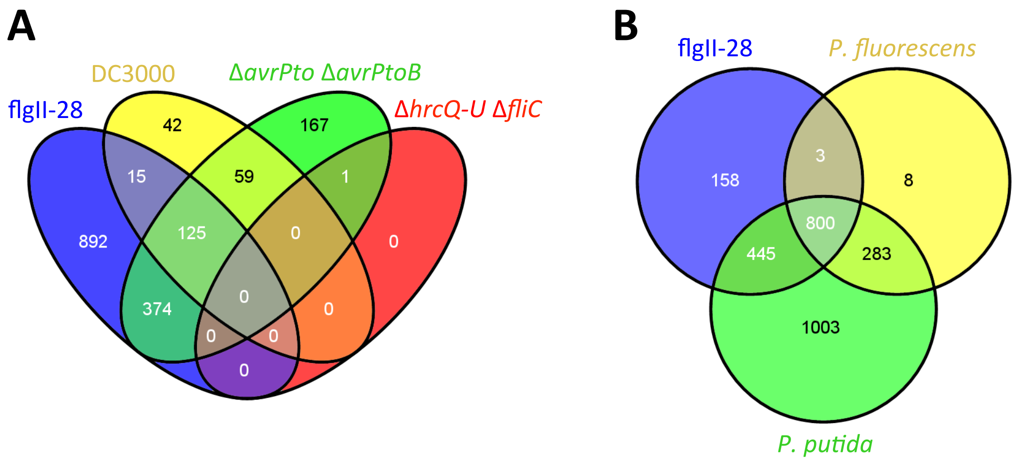

C

Strain	Total decreased	In common with flgII-28	% in common
DC3000 Δ*hrcQ-U* Δ*fliC*	1	0	0.0
DC3000	241	140	58.1
DC3000 Δ*avrPto* Δ*avrPtoB*	726	499	68.7
P. fluorescens	1094	803	73.4
P. putida	2531	1245	49.2

Additional file 3: Figure S3. Overlap between genes suppressed by bacterial strains and flgII-28 treatments. **(A)** Overlap between *Pst* strains and flgII-28; **(B)** Overlap between soil *Pseudomonas* and flgII-28. Venn diagrams were generated using Venny [2]. **(C)** Degree of overlap shown as percentage of genes.
