# Supplementary material for: Transcriptomics-based screen for genes induced by flagellin and repressed by pathogen effectors identifies a cell wall-associated kinase involved in plant immunity
Source: Genome Biol. 2013 Dec 20;14(12):R139. doi: 10.1186/gb-2013-14-12-r139 (PMC4053735; doi:10.1186/gb-2013-14-12-r139)
Supplement: Additional file 7: Figure S4 — The growth and development of SlWAK1-silenced plants are not affected. [file gb-2013-14-12-r139-S7.pdf]

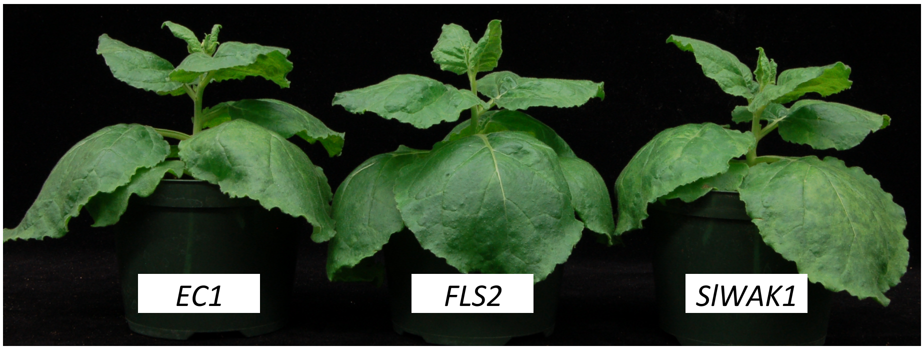

Additional file 7: Figure S4. *SlWAK1*-silenced *N. benthamiana* plants are not affected in growth or development.
