# Supplementary material for: Transcriptomics-based screen for genes induced by flagellin and repressed by pathogen effectors identifies a cell wall-associated kinase involved in plant immunity
Source: Genome Biol. 2013 Dec 20;14(12):R139. doi: 10.1186/gb-2013-14-12-r139 (PMC4053735; doi:10.1186/gb-2013-14-12-r139)
Supplement: Additional file 9: Table S5 — SlWAK1 VIGS construct target analysis in N. benthamiana. The length of 100% match regions ≥17 nucleotides long was determined by considering genes with ≥3 RPKM in at least one condition,. Predicted non-target gene information is shown at the bottom of the Table. N.C., not considered due to low or no expression. [file gb-2013-14-12-r139-S9.pdf]

Additional file 9: Table S5. *SlWAK1* VIGS construct target analysis in *N. benthamiana*. The top 10 most similar genes were identified by nucleotide BLAST [1]. Considering genes with ≥ 3 RPKM in at least one condition, the length of 100% match regions of ≥ 17 nucleotides long was determined. Predicted non-target gene information is shown at the bottom of the table. *N.C.*, not considered due to low or no expression.

Gene	Mock 6 h	*P. fluorescens* 6 h	Ratio	p value	Lengths (bp) of perfect match stretches ≥ 17 bp
NbS00011055g0005.1	0.1	6.2	88.2	0.0028	27, 30, 19, 17, 21, 20, 17, 17, 21, 24, 20
NbS00028235g0016.1	0.4	1.0	2.3	0.3847	*N.C.*
NbS00028235g0018.1	0.0	0.0	1.0	NA	*N.C.*
NbS00003635g0007.1	0.0	0.1	5.3	1	*N.C.*
NbS00011055g0002.1	0.1	5.0	67.9	8.29E-05	17, 36, 26, 17
NbS00011055g0014.1	0.2	24.8	103.1	0.0009	28, 31, 20, 18, 19, 21, 21
NbS00001112g0011.1	0.1	0.9	6.6	0.0002	*N.C.*
NbS00028235g0019.1	0.1	2.3	26.0	7.17E-13	*N.C.*
NbS00057866g0010.1	0.0	0.0	1.0	NA	*N.C.*
NbS00006163g0006.1	0.0	0.0	1.0	NA	*N.C.*
NbS00016938g0011.1	11.6	13.0	1.1	1	0

Reference

1. Altschul SF, *et al.* (1997) **Gapped BLAST and PSI-BLAST: a new generation of protein database search programs.** *Nucleic Acids Res* **25**:3389-3402.
