# Supplementary material for: Transcriptomics-based screen for genes induced by flagellin and repressed by pathogen effectors identifies a cell wall-associated kinase involved in plant immunity
Source: Genome Biol. 2013 Dec 20;14(12):R139. doi: 10.1186/gb-2013-14-12-r139 (PMC4053735; doi:10.1186/gb-2013-14-12-r139)
Supplement: Additional file 10: Figure S5 — Phylogenetic analysis of WAK and WAKL nucleotide sequences from N. benthamiana including SlWAK1, which was used to identify putative VIGS construct targets and off-targets. The PhyML method with a bootstrap of 100 replicates was used for the analysis. The bold black font indicates expressed genes with ≥3 RPKM after either mock or P. fluorescens (108 cfu/mL) treatment. Color-coded squares show the effect of P. fluorescens infiltration using a ≥2-fold difference and P < 0.05 as cut-offs. The genes considered to be possible targets of the SlWAK1 VIGS construct are in clusters A and B and are further described in Additional file 9: Table S5. The asterisk indicates the predicted VIGS non-target gene tested by qRT-PCR (Figure 5). [file gb-2013-14-12-r139-S10.pdf]

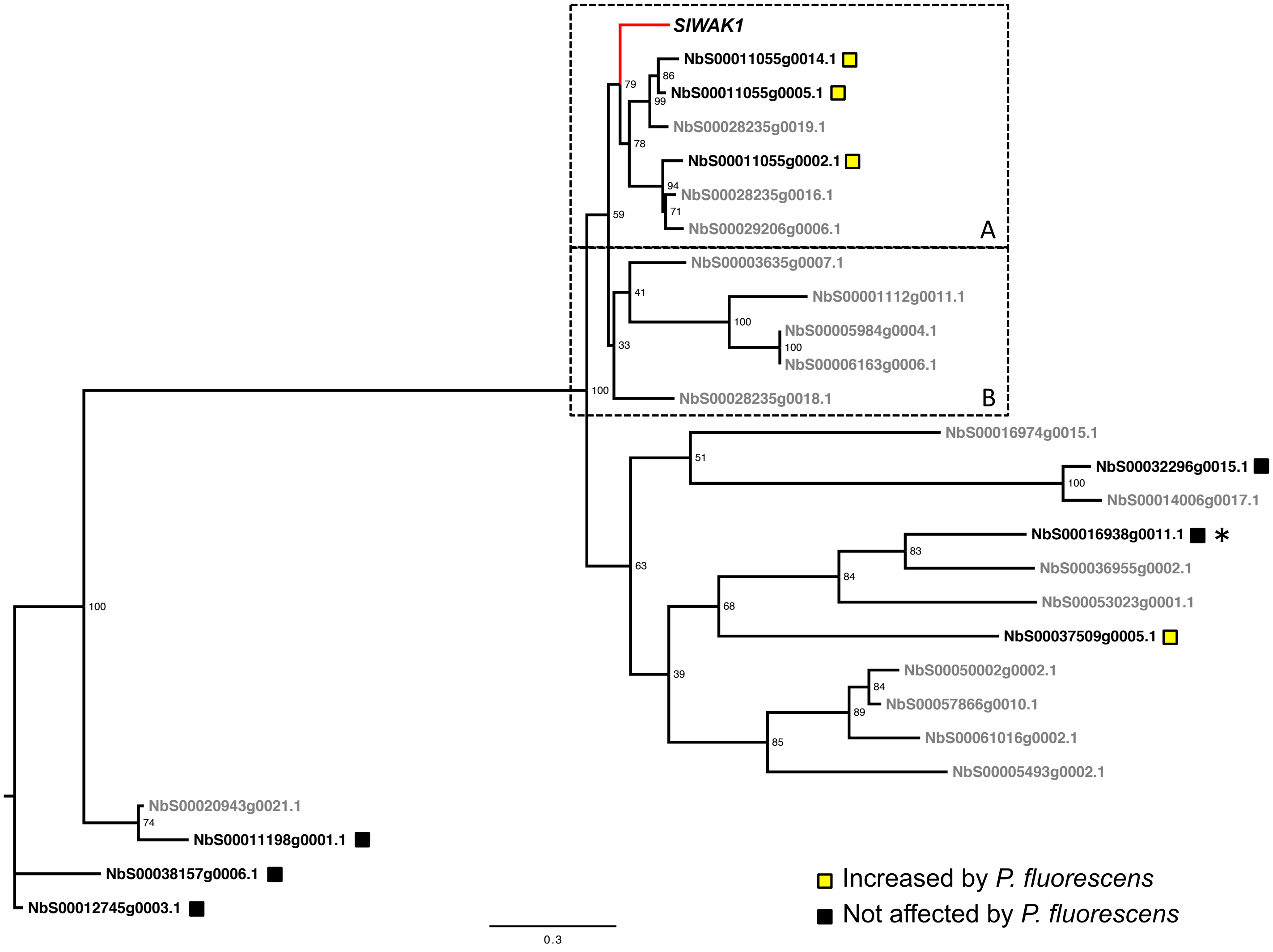

Additional file 10: Figure S5. Phylogenetic analysis of *WAK* and *WAKL* nucleotide sequences from *N. benthamiana* including *SlWAK1* for the identification of putative VIGS construct targets and off-targets. The analysis was performed with PhyML method with a bootstrap of 100 replicates using Seaview program [4]. Bold black font indicates expressed genes with ≥3 RPKM in either mock or *P. fluorescens* (10^8 cfu/mL) treatments. Color-coded squares represent the effect of *P. fluorescens* infiltration using ≥2-fold difference and a p-value <0.05 as cut-offs. The genes considered to be possible targets of *SlWAK1* VIGS construct are included in clusters A and B and further described in Additional file 9: Table S5. Asterisk indicates predicted VIGS non-target gene tested by qPCR (Figure 5).
