# Supplementary material for: Transcriptomics-based screen for genes induced by flagellin and repressed by pathogen effectors identifies a cell wall-associated kinase involved in plant immunity
Source: Genome Biol. 2013 Dec 20;14(12):R139. doi: 10.1186/gb-2013-14-12-r139 (PMC4053735; doi:10.1186/gb-2013-14-12-r139)
Supplement: Additional file 11: Table S6 — Details of the bacterial strains used in this study. [file gb-2013-14-12-r139-S11.pdf]

Additional file 11: Table S6. Details of the bacterial strains used in this study.

Strain	Characteristics[a]	Reference or source
Agrobacterium tumefaciens GV2260	Disarmed Ti plasmid; Rif^R	[1]
Escherichia coli DH5α	F– Φ80*lac*ZΔM15 Δ(*lac*ZYA-*arg*F) U169 *rec*A1 *end*A1 *hsd*R17 (rK–, mK+) *pho*A *sup*E44 λ– *thi*-1 *gyr*A96 *rel*A1	Invitrogen
Pseudomonas fluorescens 55	Wild type; Amp^R, Chl^R, Nx^R, Spc^R	[2]
Pseudomonas putida KT2440	Plasmid free derivative from strain mt-2; Amp^R	[3]
Pseudomonas syringae pv. *tomato* (*Pst*) DC3000	Wild type; Rif^R	[4]
Pst DC3000 Δ*hopQ1-1*	Δ*hopQ1-1*; Rif^R	[5]
Pst DC3000 Δ*avrPto* Δ*avrPtoB*	Δ*avrPto*::ΩSpr/Smr, Δ*avrPtoB*:::nptII, Rif^R Spc^R/Str^R Kan^R	[6]
Pst DC3000 Δ*hrcQ-U* Δ*fliC*	Δ*hrcQ-U* Δ*fliC*	[7]

[a] Rif: rifampicin; Amp: ampicillin; Chl: chloramphenicol; Nx: norfloxacin; Spc: spectinomycin; Str: streptomycin.

References

1. McBride KE, Summerfelt KR: **Improved binary vectors for *Agrobacterium*-mediated plant transformation.** *Plant Mol Biol* 1990, **14:**269-276.
2. Huang HC, Schuurink R, Denny TP, Atkinson MM, Baker CJ, Yucel I, Hutcheson SW, Collmer A: **Molecular cloning of a *Pseudomonas syringae* pv. *syringae* gene cluster that enables *Pseudomonas fluorescens* to elicit the hypersensitive response in tobacco plants.** *J Bacteriol* 1988, **170:**4748-4756.
3. Nelson KE, Weinel C, Paulsen IT, Dodson RJ, Hilbert H, Martins dos Santos VA, Fouts DE, Gill SR, Pop M, Holmes M, et al: **Complete genome sequence and comparative analysis of the metabolically versatile *Pseudomonas putida* KT2440.** *Environ Microbiol* 2002, **4:**799-808.
4. Cuppels DA: **Generation and characterization of Tn5 insertion mutations in *Pseudomonas syringae* pv. *tomato*.** *Appl Environ Microbiol* 1986, **51:**323-327.
5. Wei CF, Kvitko BH, Shimizu R, Crabill E, Alfano JR, Lin NC, Martin GB, Huang HC, Collmer A: **A *Pseudomonas syringae* pv. *tomato* DC3000 mutant lacking the type III effector HopQ1-1 is able to cause disease in the model plant *Nicotiana benthamiana*.** *Plant J* 2007, **51:**32-46.
6. Lin NC, Martin GB: **An avrPto/avrPtoB mutant of *Pseudomonas syringae* pv. *tomato* DC3000 does not elicit Pto-mediated resistance and is less virulent on tomato.** *Mol Plant Microbe Interact* 2005, **18:**43-51.
7. Wei HL, Chakravarthy S, Worley JN, Collmer A: **Consequences of flagellin export through the type III secretion system of *Pseudomonas syringae* reveal a major difference in the innate immune systems of mammals and the model plant *Nicotiana benthamiana*.** *Cell Microbiol* 2013, **15:**601-618.
