# Supplementary material for: Transcriptomics-based screen for genes induced by flagellin and repressed by pathogen effectors identifies a cell wall-associated kinase involved in plant immunity
Source: Genome Biol. 2013 Dec 20;14(12):R139. doi: 10.1186/gb-2013-14-12-r139 (PMC4053735; doi:10.1186/gb-2013-14-12-r139)
Supplement: Additional file 12: Table S7 — Summary of the sequencing data for each of the libraries generated in this work. [file gb-2013-14-12-r139-S12.pdf]

Additional file 12: Table S7. Summary of the sequencing data for each of the libraries generated in this work

Treatment	Replicate	Total reads	rRNA reads	% rRNA	Clean reads	Mapped reads	% Mapped
Mock for PAMPs treatments	1	13,571,655	212,132	1.6	13,359,523	12,548,026	93.9
	2	15,417,927	204,565	1.3	15,213,362	14,007,140	92.1
	3	21,317,819	436,538	2.0	20,881,281	19,558,297	93.7
	4	17,363,166	81,098	0.5	17,282,068	15,621,397	90.4
	Average	16,917,642	233,583	1.4	16,684,059	15,433,715	92.5
Mock for bacterial treatments	1	9,585,185	159,866	1.7	9,425,319	8,689,628	92.2
	2	14,064,287	222,296	1.6	13,841,991	12,697,661	91.7
	3	12,349,348	126,972	1.0	12,222,376	11,293,000	92.4
	Average	11,999,607	169,711	1.4	11,829,895	10,893,430	92.1
flgII-28	1	9,030,948	86,870	1.0	8,944,078	8,235,903	92.1
	2	14,728,639	63,166	0.4	14,665,473	13,267,139	90.5
	3	11,982,949	68,370	0.6	11,914,579	9,242,733	77.6
	Average	11,914,179	72,802	0.7	11,841,377	10,248,592	86.7
DC3000	1	16,512,655	154,547	0.9	16,358,108	14,878,199	91.0
	2	14,577,695	603,693	4.1	13,974,002	12,895,839	92.3
	3	13,734,719	74,218	0.5	13,660,501	12,521,376	91.7
	Average	14,941,690	277,486	1.9	14,664,204	13,431,805	91.6
DC3000 Δ*hrcQ-U* / Δ*fliC*	1	15,946,924	114,985	0.7	15,831,939	14,782,308	93.4
	2	14,051,836	140,627	1.0	13,911,209	12,655,495	91.0
	3	15,220,262	316,618	2.1	14,903,644	14,013,410	94.0
	Average	15,073,007	190,743	1.3	14,882,264	13,817,071	92.8
DC3000 Δ*avrPto* / Δ*avrPtoB*	1	13,626,646	434,354	3.2	13,192,292	12,325,604	93.4
	2	20,900,022	429,600	2.1	20,470,422	18,841,355	92.0
	3	15,240,994	453,411	3.0	14,787,583	13,623,535	92.1
	Average	16,589,221	439,122	2.7	16,150,099	14,930,165	92.5
P. fluorescens 55	1	14,887,122	543,668	3.7	14,343,454	13,212,928	92.1
	2	15,778,445	234,417	1.5	15,544,028	14,462,149	93.0
	3	14,620,555	431,915	3.0	14,188,640	13,009,188	91.7
	Average	15,095,374	403,333	2.7	14,692,041	13,561,422	92.3
P. putida KT2240	1	13,646,378	276,534	2.0	13,369,844	12,460,404	93.2
	2	15,996,225	166,402	1.0	15,829,823	14,574,519	92.1
	3	16,987,923	294,069	1.7	16,693,854	15,684,982	94.0
	Average	15,543,509	245,668	1.6	15,297,840	14,239,968	93.1
A. tumefaciens GV2260	1	11,561,441	241,769	2.1	11,319,672	10,549,336	93.2
	2	10,421,927	109,706	1.1	10,312,221	9,415,616	91.3
	3	15,117,855	224,730	1.5	14,893,125	13,982,194	93.9
	Average	12,367,074	192,068	1.5	12,175,006	11,315,715	92.8
Untreated for PAMPs treatments	1	11,241,813	211,958	1.9	11,029,855	10,270,886	93.1
	2	16,575,641	314,484	1.9	16,261,157	14,972,418	92.1
	3	19,309,950	1,105,412	5.7	18,204,538	17,245,989	94.7
	Average	15,709,135	543,951	3.2	15,165,183	14,163,098	93.3
Untreated for bacterial treatments	1	20,598,567	165,526	0.8	20,433,041	18,674,313	91.4
	2	13,369,545	73,649	0.6	13,295,896	12,179,349	91.6
	3	16,391,196	408,798	2.5	15,982,398	15,125,353	94.6
	Average	16,786,436	215,991	1.3	16,570,445	15,326,338	92.5
Total average		14,812,443	271,315	1.8	14,541,128	13,396,483	92.0
